# An Automated Workflow for Hemodynamic Computations in Cerebral Aneurysms

**DOI:** 10.1155/2020/5954617

**Published:** 2020-06-17

**Authors:** Cosmin-Ioan Nita, Takashi Suzuki, Lucian Mihai Itu, Viorel Mihalef, Hiroyuki Takao, Yuichi Murayama, Puneet Sharma, Thomas Redel, Saikiran Rapaka

**Affiliations:** ^1^Transilvania University of Brasov, Brasov, Romania; ^2^Siemens Corporate Technology, Siemens SRL, Romania; ^3^Department of Innovation for Medical Information Technology, Research Center for Medical Science, Jikei University School of Medicine, Tokyo, Japan; ^4^Siemens Medical Solutions USA, Inc., Princeton, USA; ^5^Department of Neurosurgery, Jikei University School of Medicine, Tokyo, Japan; ^6^Siemens Healthineers GmbH, Advanced Therapies, Forchheim, Germany

## Abstract

In recent years, computational fluid dynamics (CFD) has become a valuable tool for investigating hemodynamics in cerebral aneurysms. CFD provides flow-related quantities, which have been shown to have a potential impact on aneurysm growth and risk of rupture. However, the adoption of CFD tools in clinical settings is currently limited by the high computational cost and the engineering expertise required for employing these tools, e.g., for mesh generation, appropriate choice of spatial and temporal resolution, and of boundary conditions. Herein, we address these challenges by introducing a practical and robust methodology, focusing on computational performance and minimizing user interaction through automated parameter selection. We propose a fully automated pipeline that covers the steps from a patient-specific anatomical model to results, based on a fast, graphics processing unit- (GPU-) accelerated CFD solver and a parameter selection methodology. We use a reduced order model to compute the initial estimates of the spatial and temporal resolutions and an iterative approach that further adjusts the resolution during the simulation without user interaction. The pipeline and the solver are validated based on previously published results, and by comparing the results obtained for 20 cerebral aneurysm cases with those generated by a state-of-the-art commercial solver (Ansys CFX, Canonsburg PA). The automatically selected spatial and temporal resolutions lead to results which closely agree with the state-of-the-art, with an average relative difference of only 2%. Due to the GPU-based parallelization, simulations are computationally efficient, with a median computation time of 40 minutes per simulation.

## 1. Introduction

Intracranial aneurysms are pathological disorders which consist of an abnormal dilatation of the vessel wall. In severe cases, the aneurysm may rupture, causing subarachnoid hemorrhage, which can lead to severe disability or death [1]. The incidence of unruptured aneurysms is high as it occurs in about 6% of the population; however, the rupture incidence is very low, 7.7 in 100000 cases annually [2]. Consequently, the treatment of unruptured aneurysms also has a high economic cost [3]. Due to its high incidence, it is critical to accurately identify the subset of patients with high risk of rupture and plan the treatment accordingly.

There are several treatment options available to decrease the likelihood of rupture. One possibility is to surgically clip the aneurysm at its neck, to isolate the aneurysmal dome, and to prevent bleeding [4]. Another solution consists of filling the aneurysm with thin wires that constrict the flow and initiate a thrombotic reaction leading to a complete occlusion [5]. A recently proposed approach is based on placing a flow-diverter device that reduces the flow inside the aneurysm by directing most of the flow through the main artery, and inducing intra-aneurysmal thrombosis [6–8]. To establish an accurate treatment plan and to evaluate the risk of rupture, a good understanding of aneurysm hemodynamics is required. These can be used to predict the flow before and after the implantation, i.e., to investigate hemodynamic changes and the potential benefits of different therapies and choices for the patient.

The recent advances made in medical imaging, algorithms for automatically extracting anatomical information from the images, as well as in modern computing architectures (like graphics processing units), have enabled the development of much simpler workflows relying on physics-based computational models for patient-specific hemodynamic assessment [9]. Blood flow computations, when used in conjunction with patient-specific anatomical models extracted from medical images, provide important insights into the structure and function of the cardiovascular system. These techniques have been proposed for diagnosis, risk stratification, and surgical planning [10–12].

An increasing number of researchers suggest that there is a strong link between flow-related quantities and aneurysm growth or risk of rupture. This is still a highly debated subject [13, 14], and correlations found between hemodynamic quantities and aneurysm progression are not yet conclusive, as researchers proposed different quantities. Boussel et al. [15] suggest that aneurysm growth occurs in regions of low wall shear stress. Takao et al. [16] evaluated energy loss, a pressure loss coefficient, wall shear stress, and oscillatory shear index for the prediction of rupture in a set of 100 patients, suggesting that the pressure loss coefficient may be a potential parameter for predicting the risk of rupture. Furthermore, there is a debate on whether low or high wall shear stress is contributing to aneurysm risk of rupture [17]. To this extent, further efforts on integrating personalized blood flow computations in clinical workflows are crucial for developing a unified theory on aneurysm pathophysiology.

There are important technical challenges with the large-scale adoption of CFD-based tools for the clinical hemodynamic analysis of aneurysms. The methods are computationally intensive and lead to large runtimes, which is in contradiction with the high cost pressure and the continuously decreasing time a clinician can dedicate to a patient. CFD computations are commonly performed based on a discretization of the Navier-Stokes equations, using either the finite element method (FEM), finite difference methods (FDM), or finite volume methods (FVM). Models based on implicit integration using FEM have the advantage of unconditional stability, along with the ability to easily adapt to complex anatomical structures but require significant computational resources [18–20] for the solution of the resulting set of discrete equations. To further investigate the potential link between hemodynamic quantities and aneurysm outcome, a large number of computations are required; hence, an efficient approach for simulating blood flow in patient-specific anatomical models of aneurysms is of paramount importance. Although CFD-based approaches are nowadays routinely used in medical research activities to compute hemodynamic quantities under patient-specific conditions, the only CFD-based solution currently used in clinical practice, available only as a service, is focusing on computing fractional flow reserve in coronary arteries [21].

Another possible limitation of employing CFD in clinical practice is the requirement of CFD-related expertise for performing such computations, e.g., mesh generation, defining the boundary conditions, and choosing the spatial and temporal resolutions. Typical approaches to this problem consist of employing automated local mesh refinement techniques [22–25]. This limitation has also been addressed by Seo et al. [26], where a solver implementation based on the immersed boundary method has been proposed [27]. Therein, simulations are performed on a Cartesian grid using a level-set function that is directly extracted from medical images, bypassing the need of mesh generation. Recently, on-site clinical solutions have been proposed for hemodynamic analysis, e.g., for the diagnosis of coronary artery disease [28], which are based on reduced-order modeling or machine learning, and do not require specific CFD- or hemodynamics-related expertise. No on-site solution has been proposed to date relying on three-dimensional hemodynamic modeling.

To achieve a fully automated workflow for patient-specific aneurysm hemodynamics, there are two main steps which need to be considered. The first one is the extraction of anatomical models from images, and the second one is the flow computation itself. Although extracting anatomical models is a difficult problem, there are many existing solutions, both fully and semiautomated [29]. We consider that the flow computation step is still a major challenge which needs to be addressed.

In this paper, we propose an alternative approach for further automating cerebral blood flow simulations, starting from a patient-specific anatomical model reconstructed from medical images. We use a reduced-order blood flow model for computing the initial estimates of the flow distribution in all the vessel branches, which is then used to compute an initial grid resolution and time step. Furthermore, we employ an iterative approach that, if needed, then further refines the grid resolution and time step during the simulation.

To address the computational performance challenge, we employ a GPU-accelerated implementation of the lattice Boltzmann method (LBM). In recent years, LBM has emerged as a strong alternative to traditional finite element method (FEM), finite difference methods (FDM), and finite volume methods (FVM) for modeling fluid flows [30, 31]. Unlike FEM-based solvers, LBM does not require the use of complex meshing algorithms and operates on a Cartesian lattice, greatly simplifying the preprocessing step. Further, the highly local structure of the LBM algorithm results in an impressive performance on modern parallel architectures [32]. LBM has also attracted the attention in the context of cerebral flow simulation: Chopard et al. [33] employed an open source LBM implementation [34] for studying thrombus formation in a cerebral aneurysm, Bernsdorf and Wang [35] used LBM to study flow rheology in cerebral aneurysm and Závodszky and Paál [36] performed a validation study leading to good results when comparing different LBM implementations with a finite volume solver and experimental data.

Excellent results were obtained using the herein proposed solution in a very recently published clinical research paper [37]. Therein, 338 aneurysms were analyzed based on clinical, morphological, and hemodynamic parameters determined using CFD. A lower pressure loss coefficient was identified as a significant risk factor for the rupture of small intracranial aneurysms.

## 2. Methods

### 2.1. The Lattice Boltzmann Method

The lattice Boltzmann method (LBM) emerged from the lattice gas automata and is based on a discrete representation of the linearized Boltzmann equation on a regular Cartesian grid, see equation (1). Unlike the continuum Navier-Stokes-based methods that directly act on the macroscopic quantities of the flow, LBM operates at the mesoscopic scale on the particle distribution function. The discretized Boltzmann equations is

(1) $$\begin{array}{c} \left(\frac{\partial f_{i}}{\partial t}\right)+c_{i}\cdot \nabla f_{i}=\Omega \left(f_{i}\right), \end{array}$$

where *f*_*i*_(*x*, *t*) is the probability of finding a particle at spatial location *x* and time *t*, traveling with a velocity *c*_*i*_, which belongs to a set of preselected discrete velocities. The right-hand side of equation (1) represents the collision term and models the rate at which the fluid state moves towards the thermodynamic equilibrium. There are different formulations for the collision term, the most commonly used being the Bhatnagar, Groos, and Krook (BGK) model [38]. The BGK model is formulated as a linear relaxation of the distribution functions towards an equilibrium distribution *f*^eq^ and is controlled by a relaxation factor *τ* which is directly related to the kinematic viscosity *ν* through the relation, *τ* = (*ν*/*c*_*s*_^2^) + 0.5. Here, *c*_*s*_ is the speed of sound on the lattice, which can be computed for standard isotropic Cartesian lattices as *c*_*s*_^2^ = 1/3. It is the most simple and efficient implementation, but the BGK model lacks numerical stability when *τ* approaches 0.5. This limitation has been addressed and has led to other collision models, e.g., the multiple relaxation time (MRT) model [39] and the entropic model [40].

Equation (1) is a partial differential equation where the unknown is the density function *f*_*i*_. It is typically solved using an explicit time discretization scheme based on two steps: collision (2) and streaming (3), which are applied at each grid point *x*:

(2) $$\begin{array}{c} f_{i}^{\left(\text{coll}\right)}=f_{i}\left(x,t\right)-\overset{N}{\underset{j=0}{\sum }}\Omega_{i,j}\left(f_{j}\left(x,t\right)-f_{j}^{\left(\text{eq}\right)}\left(x,t\right)\right), \end{array}$$

(3) $$\begin{array}{c} f_{i}\left(x+c_{i}\delta t,t+\delta t\right)=f^{\left(\text{coll}\right)}\left(x,t\right), \end{array}$$

where *f*_*i*_^eq^ is the equilibrium distributions and depends only on the fluid velocity *u* and density *ρ*. *Ω* is the collision matrix which contains parameters controlling the relaxation of the distributions towards the equilibrium. There are different formulations for both *f*_*i*_^eq^ and *Ω*, depending on the chosen collision model, e.g., for the BGK model *Ω* = *τI*. The macroscopic velocity *u* and pressure *P* of the fluid are related to the density functions *f*_*i*_ as follows:

(4) $$\begin{array}{c} P=\rho c_{s}^{2}=\overset{N}{\underset{i=0}{\sum }}f_{i}c_{s}^{2},\\ u=\frac{1}{\rho }\overset{N}{\underset{i=0}{\sum }}c_{i}f_{i}. \end{array}$$

The discrete velocities *c*_*i*_ are associated to a lattice structure, such that each vector *c*_*i*_ corresponds to a link connecting a node *x* with a neighboring node *x* + *c*_*i*_. The most commonly employed lattice structures for 3D fluid computations are based on 15, 19, or 27 velocities. Our implementation is based on the multiple relaxation time (MRT) collision operator and a three-dimensional 19-velocity lattice [39]. The main justification for choosing the MRT collision model is the significant improvement in numerical stability at higher Reynolds number flows, compared to the classic LBGK approach. Although cerebral blood flow typically has low Reynolds number, the presence of an aneurysm can lead to more complex flow patterns which may require significantly finer grid resolutions for LBGK-based simulations. Furthermore, Závodszky and Paál [36] performed LBM simulations on an intracranial aneurysm using different collision models and showed that the MRT model is the most accurate for this flow configuration. In the MRT model, the relaxation matrix takes the form *Ω* = *M*^−1^*SM*. The distribution functions *f* are first transformed using the matrix *M* into moments *m* = *Mf*, and the relaxation towards equilibrium is performed in the moment space using the relaxation matrix *S*. The relaxation matrix *S* is a diagonal matrix containing a relaxation parameter for each moment *m*_*i*_. Once the new moments *m* have been computed, they are transformed back to the *f* space using *M*^−1^. For a more detailed description of the MRT model and for numerical values of the relaxation parameters, we refer to [39].

For lattice nodes located near a boundary, i.e., for which a neighboring node is located outside the fluid region, there are unknown *f*_*i*_ values that are required to perform the streaming step (3). The most commonly used way to compute the unknown distributions is the bounce-back approach [41]: the unknown *f*_*i*_ are set to the values corresponding to the opposite lattice direction *f*_*i*′_, such as *c*_*i*_ = −*c*_*i*′_. This is equivalent to reversing the velocity of a particle colliding with the wall. Herein, we employ an interpolated bounce-back scheme [42] that can be used for curved walls, being able to take into account the exact location of the vessel surface between two lattice locations:

(5) $$\begin{array}{c} f_{i^{\prime }}\left(x,t+1\right)=2q_{i}f_{i}^{\left(\text{coll}\right)}\left(x,t\right)+\left(1-2q_{i}\right)f_{i}^{\left(\text{coll}\right)}\left(x-c_{i},t\right)2\alpha_{i}c_{i^{\prime }}u_{w},\;q_{i}<\frac{1}{2}, \end{array}$$

(6) $$\begin{array}{c} f_{i^{\prime }}\left(x,t+1\right)=\frac{1}{2q_{i}}f_{i}^{\left(\text{coll}\right)}\left(x,t\right)+\frac{2q_{i}-1}{2q_{i}}f_{i^{\prime }}^{\left(\text{coll}\right)}\left(x,t\right)+\frac{1}{q_{i}}\alpha_{i}c_{i^{\prime }}u_{w},q_{i}\geq \frac{1}{2}. \end{array}$$

*u*_*w*_ is the prescribed velocity and *q*_*i*_ is a factor with a value between 0 and 1 that accounts for the exact position of the wall between two lattice nodes (see below for more details). For the no-slip boundaries, i.e., the vessel wall, *u*_*w*_ is set to zero, while for the vessel inflow, it is set to match the prescribed flow rate. We emphasize that this interpolated bounce-back formulation is capable of taking into account the exact location of the boundary. Although at a fundamental level the fluid domain is approximated as a staircase-shaped volume, the surface is described as an isosurface of a continuous and smooth scalar field; therefore, the boundary surface is independent of the chosen grid resolution and its exact location is always imposed.

For the outflow boundary, the velocity is typically unknown and the pressure is imposed. In this case, the nonequilibrium extrapolation method [43] is employed, which replaces all the *f*_*i*_ values at the boundary using information extrapolated from a neighboring location:

(7) $$\begin{array}{c} f_{i}\left(x,t+\Delta t\right)=f_{i}^{\text{eq}}\left(x,t\right)+\left(1-\Omega_{i,j}\right)f_{i}^{\text{neq}}\left(x_{\text{neigh}},t\right), \end{array}$$

where *f*^neq^ = *f*_*i*_ − *f*_*i*_^eq^ is the nonequilibrium part of the distribution functions, and *x*_neigh_ is a neighboring fluid node located along the boundary surface normal.

### 2.2. Grid Generation

The vessel geometry is initially given as a surface mesh where each polygon is tagged depending on the surface to which it belongs: inlets, outlets, or vessel wall. Since LBM computations are performed on a Cartesian grid, the given mesh is voxelized and a level-set representation of the vessel geometry is obtained: a signed distance field *ϕ*(*x*) such that *ϕ*(*x*) < 0 for the inside (fluid) region of the domain and *ϕ*(*x*) > 0 for the outside (solid) region. The distance field *ϕ* is computed by mapping each node to the closest polygon on the mesh and computing the signed point-to-triangle distance. The exact distance is only required for nodes located close to the boundary, to perform the interpolations described by equations (5) and (6), i.e., for computing the *q*_*i*_ values. Hence, the exact distance is computed only for nodes located in a range of ±2*δx* on both sides of the boundary. For the rest of the domain, only the sign of *ϕ* is required, and for these nodes, the distance field is extrapolated from the boundary region range. This is an important aspect of the performance improvement aspect, since computing the exact distance for the entire domain would dramatically increase the execution time [44].

**Figure 1:**
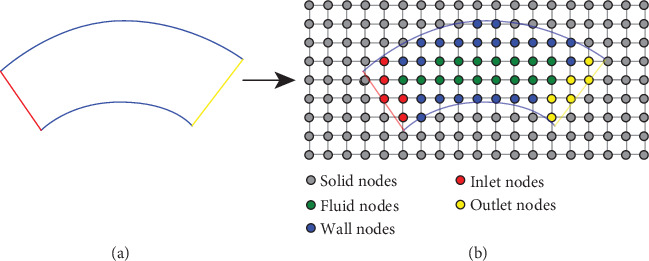
2D analogy of the node tagging process: the given surface mesh with labeled subsurfaces (a) and the grid representation (b).

The level-set function alone can be used to determine if a grid node is located at the boundary; however, additional information is required to determine which type of boundary condition to apply at each boundary node, i.e., inlet, outlet, or solid wall. Hence, each grid node needs to be labeled accordingly. The labels are computed during the voxelization step by mapping each grid node to its closest polygon on the mesh. Boundary nodes that are located at the intersection of two surfaces of different types, i.e. an inlet and wall intersection or an outlet and wall intersection, are considered corner nodes, and a special labeling logic is employed. We found the logic of labeling corner nodes to be a highly sensitive aspect, as it has a significant effect on the flow when dealing with complex-shaped boundaries. A node *x* in the grid is considered to be a boundary node if *ϕ*(*x*) ≤ 0, and there is an *i* such that *x* + *c*_*i*_ is located on the other side of the surface, i.e., *ϕ*(*x* + *c*_*i*_) > 0. A boundary node is considered to be a corner node if ∃*i*, *j*, such that the segment given by *x* and *x* + *c*_*i*_ intersects an inlet or outlet surface, and the segment given by *x* and *x* + *c*_*j*_ intersects a wall surface.  Figure 1 shows a graphical representation of the node labeling process. In the following, we present the labeling logic for each type:

1. Inlet and outlet nodes: all nodes *x* for which *ϕ*(*x*) ≤ 0, and there is an *i* such that *ϕ*(*x* + *c*_*i*_) > 0, and the segment given by *x* and *x* + *c*_*i*_ intersects an inlet or outlet surface, respectively
2. Wall nodes: all the unlabeled nodes *x* for which *ϕ*(*x*) ≤ 0, and there is an *i* such that *ϕ*(*x* + *c*_*i*_) > 0, and the segment given by *x* and *x* + *c*_*i*_ intersects a vessel wall surface
3. Bulk fluid nodes: all the remaining unlabeled nodes with *ϕ*(*x*) ≤ 0
4. Solid nodes: the remaining unlabeled nodes are labeled as solid

We emphasize that the node labeling steps must be performed sequentially, in the given order, to correctly prioritize labeling for the corner nodes.

The factors *q*_*i*_ used in equations (5) and (6) for interpolating the distributions are computed using values of the signed distance field *ϕ* at the current node *x* and the neighboring node *x* + *c*_*i*_ as follows:

(8) $$\begin{array}{c} q_{i}=\frac{\phi \left(x\right)}{\phi \left(x\right)-\phi \left(x+c\_i\right)}. \end{array}$$

All the operations described above are completely automated: after passing the initial surface mesh, there is no user interaction required for setting up the simulation. Although these operations are computationally expensive, they are only performed once in the preprocessing stage of the simulation; hence, the impact on the overall computation time is small. Under typical simulation configurations, the entire preprocessing step occupies a very small fraction of the whole computation time, for example, using a grid of 23.3 million nodes and a surface mesh of 318000 triangles, it requires around 1.4 minutes of runtime. Furthermore, the preprocessing time was found to change very little with respect to mesh size but increases quadratically when grid size is increased.

### 2.3. Automatic Model-Based Parameter Selection

Reducing the user interaction and the required CFD-related expertise represents an important aspect for employing a flow solver in a clinical setting. A key feature of our implementation is the automatic tuning of the time step *δt* and the spatial resolution *δx*, for optimizing accuracy and performance. To achieve this, we propose a heuristic approach based on the known LBM stability limits and some empirically chosen factors. More specifically, *δt* and *δx* are chosen to be as coarse as possible, but at the same time, to be small enough to capture relevant flow features and to satisfy LBM-specific stability constraints:

(9) $$\begin{array}{c} \nu_{\text{lbm}}>\nu_{\min}, \end{array}$$

(10) $$\begin{array}{c} u_{\text{lbm}}<u_{\max}, \end{array}$$

where *ν*_lbm_ and *u*_lbm_ are the nondimensional kinematic viscosity and flow velocity, respectively. For the 19-velocity MRT implementation, the critical values are *ν*_min_ = 2.54 × 10^−3^ and *u*_max_ = 0.19 [39]; however, we used smaller values, *u*_max_ = 0.15 and *ν*_min_ = 1.00 × 10^−3^, to avoid coming too close to the stability limit. These critical values are defined in terms of a lattice-based unit system, which is different from the physical viscosity and velocities. The transformation between the lattice and physical unit systems is performed using unit scale factors: *δx*, *δt*, and *δm* for position, time, and, respectively, mass. For example, the following transformations are employed for velocity *u* = *u*_lbm_(*δx*/*δt*) and pressure *P* = *P*_lbm_(*δm*/*δxδt*^2^).

Writing equations (9) and (10) for physical quantities, i.e., *ν* = *ν*_lbm_(*δx*^2^/*δt*) and *u* = *u*_lbm_(*δx*/*δt*) and collecting *δx* and *δt* leads to the stability conditions:

(11) $$\begin{array}{c} \delta t>\frac{V_{\min}}{V}\delta x^{2}, \end{array}$$

(12) $$\begin{array}{c} \delta t<\frac{u_{\max}}{\max_{x,t}\left(\left\|u\right\|\left(x,t\right)\right)}\delta x. \end{array}$$

**Figure 2:**
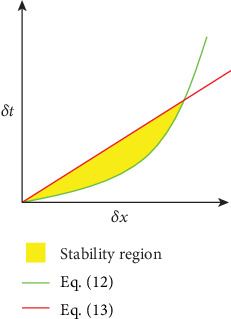
Stability region given by the LBM stability constraints for minimum viscosity (green) and for maximum velocity (red).

We emphasize that the upper threshold for the velocity *u*_max_ is expressed in lattice units, while the velocity magnitude ‖*u*‖(*x*, *t*) is expressed in physical units.  Figure 2 displays a graphical representation of the stability range, i.e., the region between the two curves. Although the lower limit of the time step, for a given spatial resolution (i.e., equation (11)), is not common for typical CFD models, for LBM, the nondimensional viscosity *ν*_lbm_ must be above the critical value *ν*_min_ which depends on the chosen collision model.

The chosen (*δx*, *δt*) values that maximize performance while remaining in the stable region are found at the upper intersection of the two curves. As for *δm*, it is computed from the physical and nondimensional density as follows:

(13) $$\begin{array}{c} \rho =\rho_{\text{lbm}}\frac{\delta m}{\delta x^{3}}. \end{array}$$

The nondimensional density *ρ*_lbm_ is typically set to 1; hence, *δm* = *ρδx*^3^. To compute the optimal (*δx*, *δt*) values using equations (11) and (12), two quantities are required: the physical kinematic viscosity *ν* and the maximum physical flow velocity *u*_max_. Viscosity *ν* is known, however, since it depends on the vessel geometry (e.g., geometries with multiple outlets), there is no information regarding the maximum velocity *u*_max_ at the beginning of the simulation. Hence, initially, *δt* and *δx* are computed using an estimated value, discussed below. As the flow is developing, the maximum flow velocity is continuously monitored, and if it exceeds the critical threshold value (equation (10)), *δt* and *δx* are adapted to satisfy the stability constraints. After a grid and/or time step refinement, the simulation is restarted from *t* = 0 using the new (*δx*, *δt*) values.

The number of required refinements (and simulation restarts) depends on the accuracy of the initial estimate of the maximum flow velocity *u*_max_. For example, if the vessel geometry presents narrowing segments, the flow velocity increases locally due to convective acceleration and a high number of refinements would be required, resulting in poor runtime performance. Similarly, if the initial estimate is too high, then the grid resolution and time step may be too fine, also resulting in poor runtime performance. A good estimate of *u*_max_ would consequently result in a good estimate of the required spatial and temporal resolutions and optimal computational performance. To improve the initial estimate of *u*_max_, we utilize a surrogate reduced-order model to estimate the distribution of flow in the vasculature. The reduced-order model is formulated as a local cross-sectionally averaged version of the Navier-Stokes equations, and therefore only solves for the total flow and pressure instead of the detailed velocity field. Such reduced-order models have been successfully used in the past to compute time-varying flow rate and pressure waveforms in full-body arterial models [45] and under pathologic conditions in specific parts of the circulation: coronary atherosclerosis [46], aortic coarctation [47], abdominal aorta aneurysm [48], and femoral bypass [49]. The one-dimensional model used herein has been previously introduced in [48] and was validated in several clinical studies [50, 51] in the context of coronary artery flow.

The one-dimensional blood flow model is derived from the three-dimensional Navier-Stokes equations based on a series of simplifying assumptions [52]. The governing equations ensuring mass and momentum conservation are

(14) $$\begin{array}{c} \frac{\partial A}{\partial t}+\frac{\partial q}{\partial x}=0,\\ \frac{\partial q}{\partial t}+\frac{\partial }{\partial x}\left(\alpha \frac{\partial q^{2}}{A}\right)+\frac{A\partial p}{\rho \partial x}=K_{R}\frac{q}{A}, \end{array}$$

where *q* = *q*(*x*, *t*) is the flow rate at the axial location *x* and time *t*, *A*(*x*, *t*) is the cross-sectional area, *p*(*x*, *t*) is the local pressure, and *ρ* is the density. Coefficients *α* and *K*_*R*_ account for the momentum-flux correction and viscous losses due to friction, respectively. A state equation is employed to close the system of equations, defining a relationship between the local cross-sectional area and the local pressure.

(15) $$\begin{array}{c} p=\Psi_{el}\left(A\right)+p_{0}=\frac{4\;Eh}{3r0}\left(x\right)\left(1-\sqrt{\frac{A_{0}}{A}}\right)+p_{0}, \end{array}$$

where *E* is the Young modulus, *h* is the wall thickness, *r*_0_ is the initial radius corresponding to the initial pressure *p*_0_, and *A*_0_ is the initial cross-sectional area. Since the purpose of using the reduced-order model is to obtain a reliable initial estimate of the flow distribution for the rigid-wall LBM model, a very large wall stiffness is chosen, i.e., the elastic parameter is set to a very large value. At each bifurcation, the continuity of flow and total pressure (sum of dynamic and statis pressure) is imposed.

The same inlet and outlet boundary conditions as for the LBM solver are employed, and the time-varying pressures and flow rates along the centerlines of the anatomical model are computed. The cross-sectional velocity profile is assumed to be parabolic; hence, the maximum velocity *u*_max_ = 2*u*_mean_ is approximated as

(16) $$\begin{array}{c} u_{\max}\approx \max\left(2\frac{q\left(x,t\right)}{A\left(x\right)}\right), \end{array}$$

where the maximum is taken for both the centerline position *x* and time *t*.

The approach of using a reduced-order model, by providing reasonable initial estimates, significantly mitigates the need for grid and time step refinement. However, since the refinement operations are affecting the total runtime performance, we apply a heuristic and reduce the initially estimated resolution by 20%, with the purpose of further improving performance. As a result, we found that only 1-2 refinement operations were needed on average for the 20 patient-specific aneurysm anatomies presented in Section 3.1.

We emphasize that the sole purpose of the reduced-order model is to provide a gross initial estimate of the required spatial and temporal resolutions, which are then further refined during the 3D simulation. Furthermore, it is not necessary for the presented 1D model to be employed for this task; any reduced-order flow model can be used, for instance [53–55].

Besides the two criteria described above for selecting the initial resolution (equations (11) and (12)), an additional criterion based on the geometry of the vessel was found to be necessary. Specifically, in cases where the vessel has a secondary branch with a very small radius and a corresponding very small flow rate, the estimated resolution may be too coarse for that branch. The additional criterion consists in limiting the *δx* value such that the minimum vessel diameter is represented by at least 15 nodes.

### 2.4. GPU Implementation

In the past, most high-performance computations were executed on large clusters of computers, each capable of executing a small number of parallel threads. However, over the last decade, general purpose graphics processing units (GPUs) have shown a tremendous increase in performance. Each GPU is capable of executing thousands of low-overhead threads simultaneously. While this kind of performance was originally developed for supporting video applications, they have become indispensable for scientific computing and for accelerating the performance of machine learning algorithms.

The lattice Boltzmann method is inherently a highly parallel algorithm, owing to the largely local nature of the computations. As discussed earlier, there are two main operations—collision and streaming. The computations in the collision step require only local information, whereas the computations in the streaming step require communication between neighboring sets of nodes. Owing to this structure of computations, advances in computing architectures, e.g., GPUs, can be exploited much better than traditional techniques such as finite element methods, which couple the solution at all nodes in the domain at each time step. Several past works have demonstrated the high performance of LBM models developed for GPU systems (for instance, [32, 56–59]).

In the following, we discuss the data structures used to optimize the LBM implementation for GPUs. Depending on the geometric complexity of the given vessel, the fluid region of the domain usually occupies a small fraction of the entire volume. Hence, allocating memory for all the nodes would be impractical and would reduce the maximum grid resolution due to memory constraint. Unstructured grids are inherently better at handling such geometric complexity, but they cannot be parallelized as easily. The main advantage of a structured grid is that one is able to access any node directly from its grid coordinates (*i*, *j*, *k*), without performing a search operation. This is an important performance aspect since, during the streaming step (3), neighboring locations *x* + *c*_*i*_ are required for each node *x*. To address this issue and at the same time maintain the advantages of a structured grid, we employed an indirect addressing scheme consisting of using an additional indexing array.

**Figure 3:**
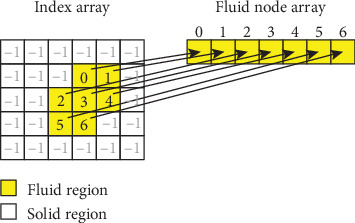
2D analogy of the indirect addressing scheme applied for handling a sparse, irregular fluid region on a Cartesian grid.

A two-dimensional analogy of the indirect addressing scheme is displayed in Figure 3. The index array contains integer indices and is used for mapping the grid coordinates (*i*, *j*, *k*) to a fluid node index. A location in the index array contains an index in the fluid nodes array or -1 if it corresponds to a solid node. Since the fluid region remains unchanged during the simulation, the content of the index array is computed only once during the preprocessing stage. The fluid node array contains the information necessary for describing the flow state: the *f*_*i*_ values, macroscopic pressures, velocities, etc. In this implementation, we have one global Cartesian grid with uniform spatial sampling. The index array is defined at every node that belongs to this Cartesian grid, containing a default value of -1 for all nodes which are outside the fluid domain, and the actual index of the fluid node for valid locations. The macroscopic variables of interest (distribution functions, velocity field, pressure, and forces) are only defined for nodes which belong to the fluid domain, resulting in significant memory savings.

As described earlier, the nodes are tagged in the initialization procedure either as belonging to the bulk of the fluid or requiring appropriate execution of a boundary condition model. Each type of node needs to be handled separately since each one may have a different implementation for the collide and streaming procedures. For instance, in the case of inflow and bounce-back nodes, the streaming step (equation (3)) is replaced by the interpolated bounce-back scheme (equation (5) and (6)), whereas for the outflow nodes the streaming step is omitted because all *f* values are completely replaced in the collision step. Therefore, an array of global indices is created in the preprocessing stage for each node type; these arrays are used to select all nodes of the same type and apply the corresponding collide and stream procedures during the simulation.

## 3. Results

### 3.1. Verification

**Figure 4:**
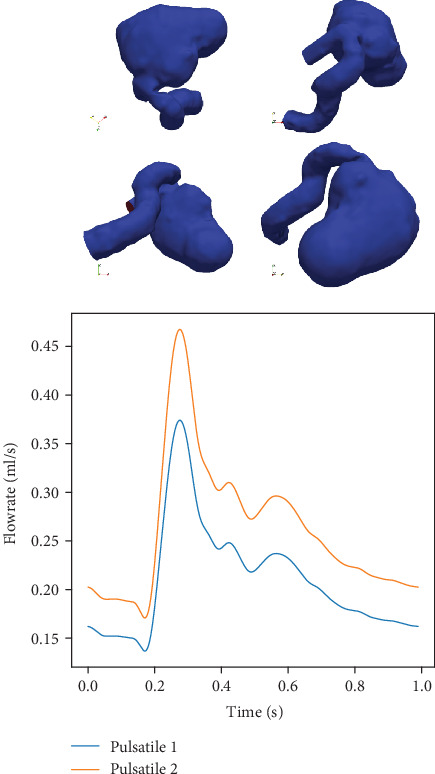
Simulation setup: the surface geometry (top) and the flow rate prescribed at the inlet boundary for the two pulsatile flows having the same waveform (bottom).

The numerical implementation of the lattice Boltzmann method was extensively validated in the past on analytical cases with known results, e.g., Womersley flow and channel flow [41, 60]. Herein, we focus on validating the methodology on real patient anatomies. First, we performed experiments on a benchmark aneurysm model previously employed in [61] as part of the “Aneurysm CFD Challenge 2012,” where the participants were required to perform CFD simulations and predict the flow. The case consists of a giant cerebral aneurysm with a proximal stenosis, displayed in Figure 4. Steinman et al. [61] reported the submitted solutions and concluded that the pressure drop caused by the stenosis was reasonably well predicted among the vast majority of the participants. We performed simulations using the same configuration and compared our results against the solutions submitted for the challenge. A time-varying flow rate was imposed at the inlet boundary, and simulations were performed for two different pulsatile flows having the same waveform (Figure 4), but different mean flow rates (5.13 and 6.41 ml/s). The spatial flow profile was flat for all simulations. At the outlet, we employed the Dirichlet boundary conditions for the pressure, imposing *p* = 0 everywhere. The initial pressure and velocity inside the fluid domain were set to zero. To reduce the transient effects that may be caused by the initial conditions, the simulations were performed for three cardiac cycles and results were extracted from the third cycle. The fluid was assumed to be Newtonian with a kinematic viscosity of *ν* = 4 mm^2^/s and a density of *ρ* = 1000 kg/m^3^.

**Figure 5:**
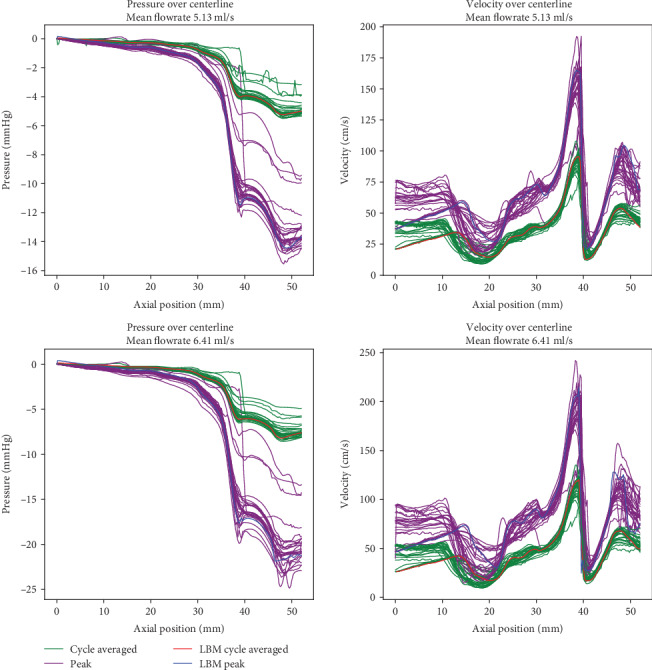
Pressures computed relative to the inlet location and velocities along the vessel centerline for the LBM simulations, and for the solutions of the CFD Aneurysm challenge [61]. Pulsatile 1 (top) and pulsatile 2 (bottom). The thick lines correspond to our solutions, while the thin ones correspond to those submitted for the challenge.

In Figure 5, we display the pressure and velocities along the centerline of the vessel computed by our model and compare them to solutions reported in [61], which correspond to solutions submitted by participants for the aneurysm challenge. The pressures in Figure 5 are computed relative to the inlet, i.e., the entire curve is shifted such that the inlet pressure is zero. Overall the centerline pressures match the published results well. As for the centerline velocities, the variability of the solutions is larger, especially in the regions close to the inlet and outlet. The main reason is that only the time-varying flow curve at the inlet was prescribed, and not the exact boundary condition to be used. This resulted in a mix of different boundary conditions, including flat profile, approximate parabolic profiles, and extensions at the inlet. Furthermore, in the outlet region, the variability is given by the turbulences that are produced by the stenosis and other geometric features of the vessel. Apart from the two regions with large variability, the LBM velocity field solutions match the aneurysm challenge solutions well. We emphasize that these computations were performed automatically, with no user interaction other than providing the surface mesh and the inlet flow rate (the spatial and temporal resolutions were automatically selected based on the approach described in Section 2).

**Figure 6:**
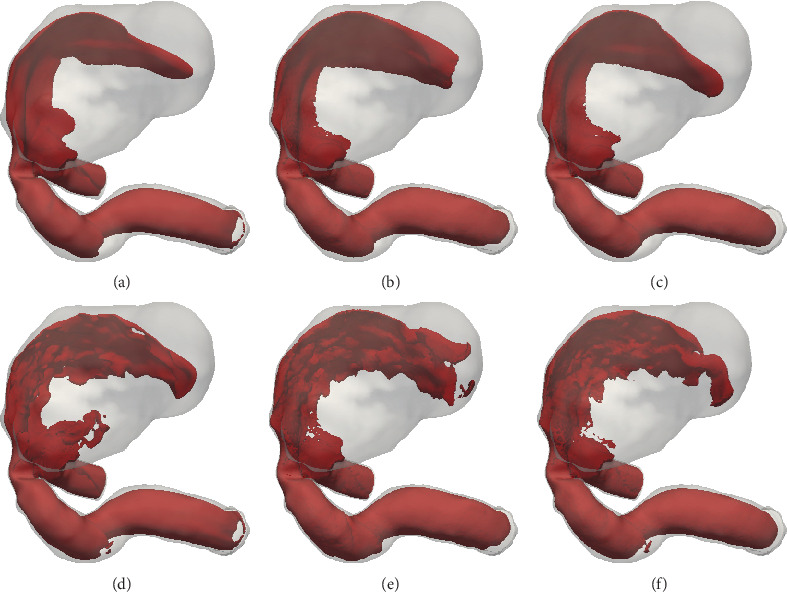
Velocity contours for the Pulsatile 2 configuration: cycle averaged velocity contours at 30 cm/s (top) and peak velocity contours at 50 cm/s (bottom) for the LBM based (a, d), Nektar1 (b, e) and Nektar2 (c, f) solutions (CFD Aneurysm challenge [61]).

In Table 1, we present the inlet-outlet pressure drop for LBM and the challenge solutions, for both pulsatile configurations. The median and the interquartile ranges are computed based on the challenge solutions. The LBM-based pressure drop values match the median values of the published solutions very well. In Figure 6, we present contour plots of the velocity field for the Pulsatile 2 configuration, for the LBM-based results, and for two solutions from [61], Nektar 1 and Nektar2, which are considered to be the reference solutions as they are based on a spectral element solver with high spatial and temporal resolutions.

To further validate our solver, we have performed simulations on 20 patient-specific aneurysm cases and compared the results against those obtained using a commercially available CFD solver (Ansys CFX, Canonsburg PA, http://www.ansys.com/). The cases correspond to ten internal carotid artery (ICA) and ten middle cerebral artery (MCA) aneurysms. A more extensive verification of our solver on aneurysm cases was performed in [62]. Simulations were performed under the same configuration as herein, three cardiac cycles, and results were extracted from the last cycle only; a time-variable velocity is specified at the inlet boundary, while the outlet is set to have constant pressure. The grid resolution was automatically estimated using the approach proposed in Section 2.

We first compared two quantities which were previously shown to be important indicators for the risk of rupture in aneurysms: the pressure loss coefficient (PLc) and the average wall shear stress on the aneurysm dome (AvWSS) [16]. The pressure loss coefficient is a nondimensional quantity describing the relative pressure drop and is defined as follows:

(17) $$\begin{array}{c} \text{PLc}=\frac{\left(\left(1/2\right)\rho u_{\text{in}}^{2}+P_{\text{in}}\right)-\left(\left(1/2\right)\rho u_{\text{out}}^{2}+P_{\text{out}}\right)}{\left(1/2\right)\rho u_{\text{in}}^{2}}, \end{array}$$

where *P*_in_ and *P*_out_ are the mean pressures measured at the inlet and outlet planes, respectively, while *u*_in_ and *u*_out_ are mean velocities measured at the same planes. The inlet and outlet planes used for computing PLc are placed perpendicular to the vessel centerline at approximately 1 mm before, and, respectively, after the aneurysm.

WSS is typically computed using spatial velocity gradients; however, using MRT-LBM, the WSS can be extracted directly from the nonequilibrium moments *m*^neq^ = *m* − *m*^eq^, as described in [63].

**Figure 7:**
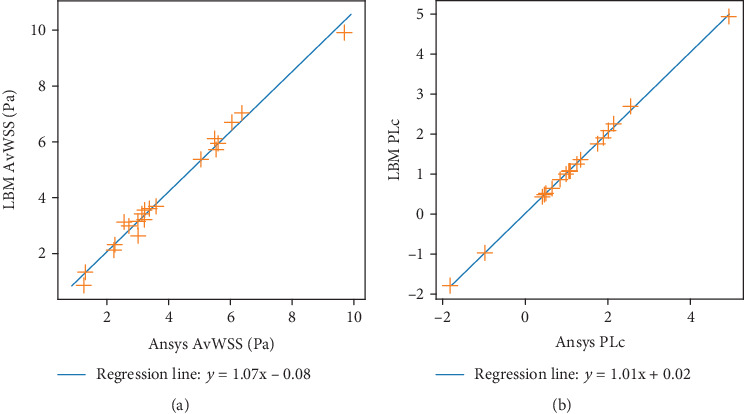
Comparison between LBM and Ansys CFX for pressure loss coefficient (b) and average WSS (a) on the aneurysm dome for all cases.

In Figure 7, we present the comparison for both PLc and AvWSS. Correlation between Ansys CFX and our proposed implementation appears to be exceptionally good for both quantities: the Pearson correlation was 0.999 and 0.993 for PLc and AvWSS, respectively, while the *p* value was 0 for both cases.

A quantitative comparison of the centerline pressures and velocity magnitude was performed for all twenty cases, and the results are displayed in Tables 2 and 3. Since most of the cases have multiple outlet branches, the comparison was performed for each branch separately. The centerline curves contain between 500 and 1000 points, and the differences were taken at each point and represented relative to the CFX quantity averaged over the entire centerline. More specifically, at each point on the centerline, the absolute difference was divided by the average CFX pressure or velocity:

(18) $$\begin{array}{c} d\left(x\right)=\frac{\;|\;f_{\text{LBM}}\left(x\right)-f_{\text{CFX}}\left(x\right)\;|\;}{{\bar{f}}_{\text{CFX}}}\cdot 100\%, \end{array}$$

where *d* is the relative difference, *x* is the position along the centerline, and *f* is the quantity of interest, i.e, pressure/velocity. The average relative difference was 2.1% and, respectively, 2.21% for peak systole and cycle averaged pressure, and 1.85% and 1.73%, respectively, for the velocity.

Furthermore, we computed the total pressure drop for all cases, both for the LBM-based and CFX results. Since the outlet pressure was always set to zero, the total pressure drop corresponds to the inlet pressures. Using these values, the relative difference between LBM and CFX was computed. The maximum difference was found to be 4.5% for the cycle averaged pressure and 5.14% for the peak pressure, corresponding to the MCA9 case, while the minimum differences were 0.05% and 0.36% for the cycle averaged and peak pressures, respectively, corresponding to the ICA6 case. On average, the relative differences were 2.15% and 2.5% for cycle averaged and peak systole quantities.

**Figure 8:**
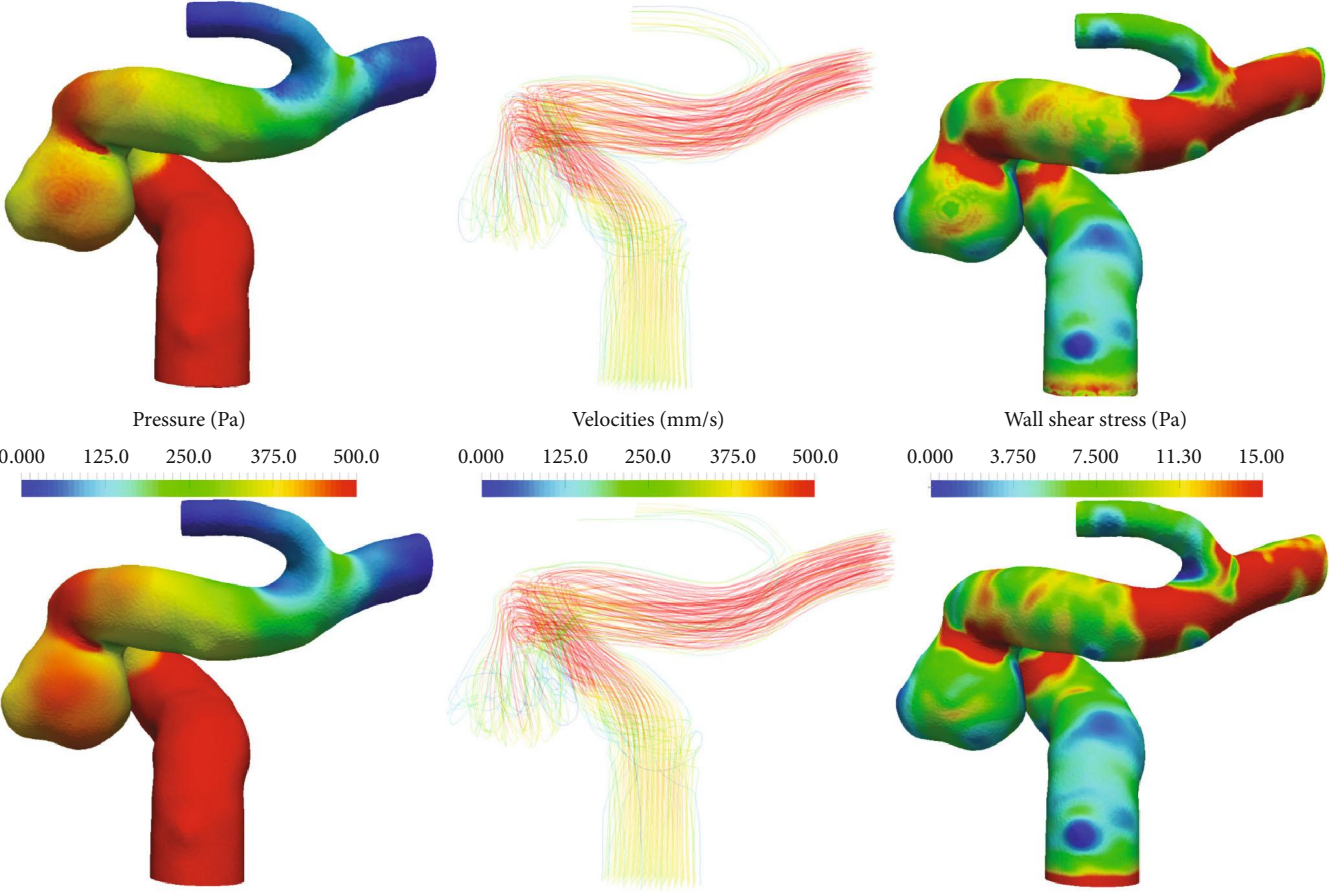
Pressures, velocities, and wall shear stress for the ICA1 case at peak systole. Simulation results are based on LBM (first row) and Ansys CFX (second row).

**Figure 9:**
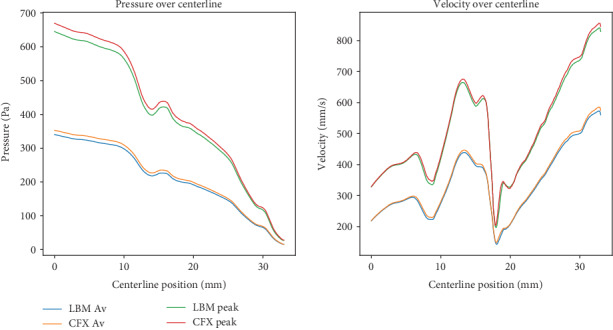
Pressures and velocities along the centerline, corresponding to the main branch of the ICA1 case.

A visual comparison of the results corresponding to one of the cases is provided in Figures 8 and 9.  Figure 8 presents the pressure, velocity, and wall shear stress (WSS) fields for both the LBM-based and CFX results, and Figure 9 presents the pressure and velocity plot along the centerline of the main branch for the same case.

Since LBM is based on an explicit discretization scheme, there is an implicit delay in the flow propagation from inlet to outlet. In our experiments, we found the delay to be about 20 ms for all cases and outlets. Although this is an undesired aspect, there is a certain delay in physiological conditions caused by wall elasticity. Unfortunately, an accurate quantification of this physiological delay is currently not possible.

Overall, the LBM results appear to match well the CFX results for both cycle averaged and peak systole quantities, without requiring any human intervention in selecting an appropriate grid resolution.

### 3.2. Convergence Study

To demonstrate that the automatically selected grid size is sufficiently fine, we have performed a convergence study on two representative cases, i.e., an ICA and a MCA case. We performed computations with five different values of the spatial resolution: *δx*/2, *δx*, 2*δx*, 3*δx*, and 4*δx*, where *δx* is the automatically selected value. Since the computed spatial resolution and time step are typically chosen such that they are close to the stability limit, as described in Section 2.3, it would normally not be possible to perform simulations with coarser resolutions (2*δx*, 3*δx*, and 4*δx*) because of the chosen stability limits. Therefore, to perform these simulations, we set the stability for *ν*_min_ and *u*_max_ to the original values from [39]: *u*_max_ was increased to 0.19, and *ν*_min_ was decreased to 2.56*e*-3. Also, the third criterion (*D*_min_/*δx* > 15) was removed.

**Figure 10:**
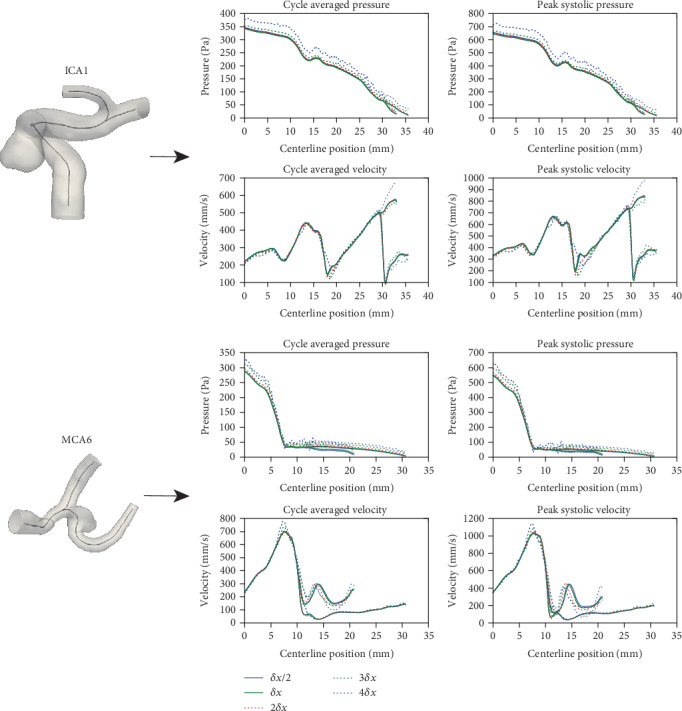
Convergence analysis was performed on an ICA case and a MCA case. The plots display peak systole and cycle averaged pressures and velocities along the centerlines. Bifurcations on the plot correspond to the bifurcation near the outlets. Green lines on each plot correspond to simulations performed using the automatically selected spatial resolution value *δx*, the blue line corresponds to a grid two times finer (*δx*/2), while the others correspond to coarser grids.

Figure 10 displays the results as plots of the pressure and velocity magnitude along the vessel centerline. The green and blue plots correspond to the automatically selected, and, respectively, a twice as fine spatial resolution; the other plots correspond to coarser resolutions. The convergence trend is well visible for both cases. All results were compared against the reference values corresponding to the finest resolution (*δx*/2).  Table 4 displays for each case the mean absolute errors as a function of the spatial resolution. For the simulations corresponding to the automatically selected spatial resolution (first column of Table 4), the maximum error relative to the mean quantity along the centerline is 2.18%. We emphasize that the purpose of these experiments is to show that running the computations at a finer resolution will not significantly change the results, therefore indicating that the automatically chosen resolution is sufficiently fine.

### 3.3. Computational Efficiency

All LBM computations were performed on a regular workstation with an NVIDIA GTX 1080ti graphics card. The GPU implementation was based on the NVIDIA CUDA version 8.0, and all computations were performed using double precision arithmetic. In Table 5 we report the LBM computation time for each case along with the corresponding grid size and time step. We emphasize that *δx* and *δt* were chosen automatically using the approach described in Section 2. The CFX simulations were performed on a cluster of three nodes of 12 CPU cores each. The mesh sizes ranged from 2.6 to 11.3 million tetrahedral elements and were chosen after a grid convergence study. For the flow configuration presented in this paper, the LBM computation time was found to vary between 10 minutes and 300 minutes, which is significantly faster than the typical runtimes reported using existing methods in literature [18, 19]. We have only used a single, commodity GPU on standard workstation for the computations. The grid sizes varied between 50 *μ*m and 120 *μ*m, and the time step between 3 *μ*s and 23 *μ*s.

## 4. Discussion

Performing CFD computations is typically a challenging task, especially for complex flows like in the case of cerebral aneurysms. The main challenges are given by computational complexity, which leads to large execution times, but also by the requirement of having an experienced user for choosing solver parameters, mesh resolution, etc. Both of these are limiting factors that reduce the potential of employing CFD-based tools in clinical settings, where patient-specific computations need to be performed. Herein, we have addressed these limitations and proposed a novel methodology for performing hemodynamic computations in patient-specific cerebral aneurysms. The computational cost was significantly reduced by employing a GPU-based implementation of the lattice Boltzmann method. A CFD simulation for one cerebral aneurysm can be performed in a matter of minutes on a regular workstation compared to hours on expensive computing clusters. We performed computations on 21 aneurysm cases and the median execution time was 40 minutes using a single-commodity GPU. We emphasize that the measured time includes the preprocessing step, all three simulated cardiac cycles, and also the simulation restarts required for tuning the spatial resolution and the time step. Although the computation time may still be considered too high for employing such tools in a clinical setting, it can be significantly reduced by further increasing the parallelism, e.g., by using multiple GPUs simultaneously [32]. Furthermore, we found that there is a strong dependence between computation time and vessel geometry complexity, i.e., narrowing segments, curvature, and branching. As the flow develops more complex features, a finer resolution is required, which increases the computation time. Also it could be argued that real-time performance may not be necessary for aneurysmal flow computation in clinics as there is no reason for results to be obtained synchronously. However, performance could become important in a virtual treatment scenario where a clinician may want to explore the possibility of deploying an endovascular device and change different properties. Also computational performance could become important for other cardiovascular diseases where flow-related quantities are needed immediately, e.g., coronary artery disease.

We showed that even though performance is significantly improved, the accuracy is not affected. We extracted the results from all simulations and compared them with other publicly available solutions and also with a commercial solver. First, we considered the solutions reported for a CFD challenge [61], which contains multiple simulation results obtained using various flow solvers and configurations. The comparison shows an exceptionally good agreement, as our solutions lies very close to median of the others, as indicated in Table 1. Furthermore, we performed a comparison of the velocity contours with two of the solutions reported in [61], considered to be the best resolved ones. Although the flow presents strong turbulences inside the aneurysm dome, the velocity contours appear to match very well. Lastly, we compared results with those obtained with a commercial solver (Ansys CFX) on 20 aneurysm cases. A good agreement between solutions was found, with an average relative difference of 2%. We emphasize that for all validation experiments, our results were automatically obtained using the proposed methodology, with no user interaction other than providing the vessel surface mesh. We further emphasize that this comparison is by no means a definitive process for evaluating the accuracy of our results; however, currently, there are no available methods for accurately measuring flow-related quantities in vivo.

Furthermore, since the workflow is completely automated, a clinician may use such a tool without requiring CFD-related experience and expertise. To demonstrate that the automatically chosen grid size and time step are sufficiently fine, we performed a convergence analysis on two randomly chosen cases, by running simulations with both finer and coarser spatial resolutions. We found that a spatial resolution twice as fine led to a maximum of only 2.18% relative change in centerline pressure and velocity.

To formulate the criteria for grid refinement, we start from the stability constraints implicitly present in the LBM method, in combination with the prior information obtained through the reduced-order blood flow model. More rigorous criteria for grid refinement were also proposed in the past, for example, Axner et al. [64] proposed a criterion for cases where the flow is driven by a Womersley profile; similarly, Lagrava et al. [23] proposed a criterion for performing automatic grid refinement with LBM. Although we employed a rather heuristic approach, it is based on the same fundamental aspect: the error is proportional to the nonequilibrium part of LBM distribution functions.

As for the limitations of the proposed workflow, the most important one is the lack of patient-specific information regarding the boundary conditions used for the simulations, especially the time-varying flow rate and its profile imposed at the inlet boundary, the outflow resistances, and the viscosity models. Although these are important parameters, other studies indicate that flow quantities are much more sensitive to geometry than to flow parameters, i.e., changes in the anatomical model of the vessel, obtained as a result of the segmentation and reconstruction process, have the greatest impact on accuracy [65–69]. As further research will shed light on the relevant flow-related quantities having the greatest impact on aneurysm pathophysiology, progress will be made towards a unified modeling technique for aneurysmal flow, e.g., by improving the accuracy of the relevant flow aspects and determining which modeling methodology performs best.

We would like to emphasize that the purpose of including Ansys CFX in the verification process is solely for quantifying the accuracy of our results, by comparing them with a trusted state-of-the-art method which is generally accepted in the community. By no means, do we consider our implementation (or LBM methods in general) to be superior for this class of problems in terms of efficiency or other aspects.

## 5. Conclusions

We proposed a workflow aimed at improving the potential of using CFD-based tools in a clinical setting, as a tool for aiding decision making and establishing a personalized treatment plan for cerebral aneurysms. We addressed the main limitations: the requirement of CFD-related experience and the computational performance. To speed up the CFD computations, we employed a GPU-accelerated flow solver based on the lattice Boltzmann method. We showed that for this particular flow configuration, the computation time was reduced to minutes on a regular workstation, whereas typical CFD computation times are much larger even when performed on expensive computing clusters. We introduced a fully automatic pipeline for selecting the mesh resolution and time step using solely vessel geometry information, allowing thus clinicians to perform computations and obtain results with minimal CFD-related expertise. We showed that even though computation time was greatly reduced, there is no significant impact on accuracy. We performed computations on several aneurysm cases and compared results with other publicly available solutions and also with a commercial solver. An average relative difference of 2% between the solutions was found. Furthermore, a convergence study was performed, showing that the automatically chosen spatial resolution is sufficiently fine for the chosen case.

Future work will focus on further adapting the automated parameter selection process to also enable the inclusion of flow diverters in the simulation. We also consider further extending the workflow for a broader range of blood flow computations, e.g., the coronary arteries and aorta.

## Figures and Tables

**Table 1:** Inlet pressure drop for the solutions in [61] and for the LBM-based approach.

Case	Steinman phase I CFD solutions		LBM
	Median	Interquartile range	
Pulsatile 1			
Peak	13.7	13.0-13.9	14.2
Cycle averaged	5.0	4.8-5.1	5.0
Pulsatile 2			
Peak	20.5	19.4-21.0	20.5
Cycle averaged	7.6	7.2-7.8	7.7

**Table 2:** Comparison between centerline velocity magnitudes obtained with LBM and Ansys CFX. The differences are expressed in percentages relative to the CFX velocity at each point on the centerline and are computed separately for each outlet branch of the vessel.

Case	Branch	Cycle average		Peak systole	
		Median	Interquartile range	Median	Interquartile range
IC1	0	-1.79	(-2.30, -1.31)	-1.58	(-2.20, -1.03)
	1	-2.04	(-2.56, -1.41)	-1.80	(-2.83, -1.13)
IC2	0	-2.02	(-3.23, -1.42)	-2.58	(-3.74, -1.38)
	1	-1.67	(-2.33, -1.21)	-1.90	(-2.65, -0.92)
IC3	0	-0.29	(-0.40, 0.19)	-0.03	(-0.16, 2.72)
	1	-0.38	(-1.83, -0.13)	-0.14	(-2.91, 0.06)
IC4	0	-1.82	(-2.25, -1.15)	-1.26	(-2.58, -0.70)
IC5	0	-0.43	(-0.87, -0.18)	-0.52	(-0.88, -0.08)
	1	-0.66	(-1.05, -0.42)	-0.68	(-1.19, -0.39)
IC6	0	-1.70	(-2.45, -1.05)	-2.17	(-3.10, -0.80)
	1	-1.28	(-1.91, -0.50)	-2.35	(-3.81, -1.06)
IC7	0	-0.65	(-0.95, -0.54)	-0.60	(-0.89, -0.30)
	1	-0.48	(-0.65, -0.23)	-0.27	(-0.64, -0.05)
	2	-0.57	(-0.75, -0.26)	-0.43	(-0.78, -0.16)
IC8	0	-0.93	(-1.45, -0.61)	-0.68	(-1.33, -0.33)
	1	-0.90	(-1.41, -0.60)	-0.64	(-1.25, -0.30)
	2	-1.09	(-1.61, -0.75)	-0.79	(-1.51, -0.41)
IC9	0	-0.89	(-1.60, -0.26)	-0.71	(-1.90, -0.17)
IC10	0	-2.02	(-2.43, -1.33)	-1.79	(-2.50, -1.20)
	1	-1.94	(-2.39, -1.05)	-1.71	(-2.44, -1.12)
	2	-3.17	(-4.32, -2.37)	-2.73	(-3.37, -1.90)
MCA1	0	-1.46	(-2.73, -0.86)	-1.18	(-2.18, -0.59)
	1	-0.66	(-1.10, -0.47)	-0.61	(-1.24, -0.22)
	2	-2.31	(-3.60, -1.35)	-1.33	(-1.78, -0.79)
MCA2	0	-1.23	(-1.62, -0.73)	-0.98	(-1.51, -0.61)
	1	-1.59	(-2.12, -1.07)	-1.74	(-3.04, -1.03)
MCA3	0	-2.06	(-2.73, -0.86)	-2.09	(-2.90, -0.80)
	1	-4.07	(-7.38, -2.57)	-3.47	(-6.41, -2.19)
MCA4	0	-0.59	(-1.98, 0.14)	-1.21	(-2.11, -0.28)
	1	-2.82	(-4.12, -2.19)	-3.02	(-4.05, -1.96)
MCA5	0	-2.56	(-3.01, -2.27)	-2.40	(-3.09, -2.15)
	1	-2.50	(-2.91, -1.56)	-2.40	(-2.76, -1.61)
	2	-1.03	(-3.27, -0.80)	-1.43	(-2.69, -1.09)
MCA6	0	-2.15	(-3.23, -0.57)	-2.30	(-3.70, -1.03)
	1	2.78	(-2.13, 3.47)	2.00	(-2.07, 2.62)
MCA7	0	-2.87	(-3.92, -2.25)	-2.92	(-3.81, -1.95)
	1	-2.90	(-4.02, -2.25)	-2.90	(-3.96, -2.19)
	2	-3.64	(-4.14, -2.42)	-3.49	(-4.26, -2.18)
MCA8	0	-3.87	(-4.63, -3.02)	-3.62	(-4.37, -2.60)
	1	-2.50	(-4.24, 0.00)	-2.09	(-4.15, -0.58)
	2	-2.32	(-4.33, -0.03)	-1.90	(-4.22, -0.50)
	3	-3.69	(-4.40, -2.16)	-3.01	(-4.25, -1.72)
MCA9	0	-1.78	(-3.14, -1.32)	-1.55	(-2.55, -1.12)
	1	-2.60	(-3.29, -1.78)	-2.39	(-3.46, -1.14)
	2	-1.81	(-3.24, -1.64)	-1.83	(-2.89, -1.55)
	3	-1.33	(-3.63, -0.94)	-1.17	(-3.02, -0.65)
MCA10	0	-2.07	(-3.31, -0.86)	-1.86	(-3.00, -0.71)
	1	-2.72	(-3.24, -1.95)	-2.64	(-3.24, -1.61)
	2	-2.25	(-3.31, -1.22)	-2.13	(-3.13, -1.16)
Overall (mean of absolute values)		1.85		1.73	

**Table 3:** Comparison between centerline pressures obtained with LBM and Ansys CFX. The differences are expressed in percentages relative to the CFX pressure at each point on the centerline and are computed separately for each outlet branch of the vessel.

Case	Branch	Cycle average		Peak systole	
		Median	Interquartile range	Median	Interquartile range
IC1	0	-3.98	(-5.33, -2.29)	-4.11	(-5.47, -2.43)
	1	-3.92	(-5.48, -2.14)	-4.13	(-5.64, -2.31)
IC2	0	-3.45	(-5.39, -0.94)	-3.58	(-5.65, -1.13)
	1	-3.81	(-4.99, -1.62)	-3.90	(-5.23, -1.61)
IC3	0	-1.50	(-3.58, -0.74)	-2.26	(-3.91, -0.47)
	1	-1.20	(-3.82, -0.45)	-2.14	(-4.20, -0.63)
IC4	0	-0.57	(-1.85, 1.05)	-2.25	(-3.50, -0.59)
IC5	0	0.13	(0.00, 0.69)	-0.22	(-0.40, 0.36)
	1	0.08	(0.00, 0.44)	-0.25	(-0.36, 0.05)
IC6	0	1.28	(0.76, 2.54)	0.53	(0.16, 1.45)
	1	1.29	(0.78, 1.80)	0.23	(-0.43, 0.86)
IC7	0	-0.67	(-0.79, -0.44)	-0.62	(-0.72, -0.40)
	1	-0.58	(-0.86, -0.38)	-0.52	(-0.76, -0.29)
	2	-0.51	(-0.89, -0.26)	-0.45	(-0.79, -0.10)
IC8	0	-0.24	(-1.08, 1.48)	-0.61	(-1.45, 1.01)
	1	-0.19	(-1.10, 1.64)	-0.58	(-1.49, 1.14)
	2	-0.74	(-0.90, -0.31)	-0.98	(-1.16, -0.54)
IC9	0	-5.14	(-5.36, -4.58)	-5.92	(-5.99, -5.62)
IC10	0	-2.09	(-4.13, 0.14)	-2.42	(-4.71, 0.25)
	1	-2.34	(-3.47, -1.30)	-3.01	(-3.96, -1.39)
	2	-0.68	(-3.33, 1.78)	-0.36	(-4.09, 1.19)
MCA1	0	0.22	(-0.44, 2.74)	-0.74	(-1.35, 1.49)
	1	-0.11	(-0.53, 0.73)	-1.17	(-1.59, 0.48)
	2	2.59	(-0.40, 3.91)	1.35	(-1.41, 2.18)
MCA2	0	-0.96	(-1.98, 0.51)	-1.78	(-2.90, -0.13)
	1	-0.89	(-1.59, -0.27)	-1.52	(-2.43, -0.70)
MCA3	0	-4.70	(-6.11, -1.99)	-4.67	(-6.53, -2.07)
	1	-3.21	(-5.39, 0.84)	-3.39	(-5.69, 0.14)
MCA4	0	-0.49	(-0.72, -0.08)	-1.35	(-1.78, -1.09)
	1	1.28	(-0.05, 5.90)	-0.12	(-1.53, 4.61)
MCA5	0	-1.17	(-1.89, 0.35)	-1.75	(-2.55, -0.15)
	1	-0.91	(-1.90, 0.45)	-1.54	(-2.60, -0.08)
	2	-1.38	(-2.11, -0.63)	-2.01	(-2.89, -0.89)
MCA6	0	5.48	(-0.95, 8.75)	3.97	(-1.95, 7.47)
	1	5.21	(2.73, 6.46)	3.62	(2.09, 4.62)
MCA7	0	-4.29	(-5.57, -2.19)	-5.31	(-6.62, -2.91)
	1	-3.95	(-5.44, -2.18)	-4.94	(-6.47, -2.92)
	2	-4.19	(-5.39, -3.06)	-5.07	(-6.37, -3.88)
MCA8	0	1.14	(-2.00, 4.33)	-0.13	(-3.58, 3.30)
	1	1.13	(-2.00, 1.32)	-0.03	(-3.53, 0.31)
	2	0.99	(-1.83, 1.33)	-0.05	(-3.38, 0.21)
	3	1.16	(-1.94, 1.78)	-0.03	(-3.43, 0.60)
MCA9	0	-5.04	(-5.39, -1.10)	-5.10	(-5.41, -1.20)
	1	-3.95	(-5.63, -0.59)	-4.14	(-5.71, -0.64)
	2	-2.50	(-5.58, -1.64)	-2.50	(-5.78, -1.69)
	3	-3.70	(-5.39, -2.14)	-3.63	(-5.43, -2.17)
MCA10	0	-2.50	(-4.72, -2.23)	-2.97	(-5.48, -2.54)
	1	-2.96	(-5.30, -1.13)	-3.66	(-6.26, -1.56)
	2	-2.55	(-4.71, -2.13)	-3.02	(-5.47, -2.43)
Overall (mean of absolute values)		2.10		2.21	

**Table 4:** Mean absolute errors of the results as a function of the spatial resolution *δx*. ‘Av' and ‘Pk' entries in the third column stand for cycle averaged and peak systolic, respectively.

Quantity	Case		*δx*	2*δx*	3*δx*	4*δx*
Pressures (Pa)	ICA1	Pk	1.81	7.60	17.93	27.13
		Av	2.32	2.59	10.80	31.35
	MCA6	Pk	2.28	9.58	22.60	34.19
		Av	2.15	9.00	18.02	24.03
Velocities (mm/s)	ICA1	Pk	0.79	1.95	4.54	6.72
		Av	2.10	4.35	8.62	16.90
	MCA6	Pk	2.47	6.10	14.18	20.97
		Av	4.17	13.38	26.89	32.37

**Table 5:** Execution times and grid size for each case.

Case	*dx* (mm)	*Dt* (s)	Grid size (only fluid nodes)	Execution time (min)
CFD challenge				
Pulsatile 1	0.14	9.04*e*-6	12.1*e*+6	44.6
Pulsatile 2	0.11	5.7*e*-6	23.3*e*+6	123.4
ICA1	0.084	1.03*E*-05	6.23*E*+05	26.85
ICA2	0.080	9.90*E*-06	6.70*E*+05	35.89
ICA3	0.069	8.54*E*-06	1.62*E*+06	92.73
ICA4	0.093	1.43*E*-05	3.96*E*+05	22.35
ICA5	0.052	3.25*E*-06	3.32*E*+06	280.16
ICA6	0.115	1.77*E*-05	5.46*E*+05	22.45
ICA7	0.057	7.03*E*-06	6.74*E*+06	298.91
ICA8	0.090	8.83*E*-06	7.74*E*+05	36.08
ICA9	0.118	2.28*E*-05	2.26*E*+05	9.99
ICA10	0.093	1.43*E*-05	1.03*E*+06	76.40
MCA1	0.051	7.89*E*-06	3.19*E*+06	188.87
MCA2	0.053	8.21*E*-06	1.79*E*+06	129.87
MCA3	0.082	1.58*E*-05	4.87*E*+05	27.42
MCA4	0.091	1.75*E*-05	4.67*E*+05	20.18
MCA5	0.059	1.13*E*-05	6.95*E*+05	49.10
MCA6	0.078	4.99*E*-02	4.74*E*+05	43.91
MCA7	0.082	1.59*E*-05	3.46*E*+05	35.88
MCA8	0.079	1.52*E*-05	3.10*E*+05	20.07
MCA9	0.038	5.91*E*-06	3.22*E*+06	252.09
MCA10	0.070	1.07*E*-05	5.95*E*+05	43.91

## Data Availability

The patient data used to support the findings of this study were supplied by Department of Innovation for Medical Information Technology, Research Center for Medical Science, Jikei University School of Medicine, Tokyo, Japan, so cannot be made freely available.
